# Investigation of the Microstructures of Graphene Quantum Dots (GQDs) by Surface-Enhanced Raman Spectroscopy

**DOI:** 10.3390/nano8100864

**Published:** 2018-10-22

**Authors:** Junxiao Wu, Peijie Wang, Fuhe Wang, Yan Fang

**Affiliations:** The Beijing Key Laboratory for Nano-photonics and Nano-structure, Department of Physics, Capital Normal University, Beijing 100048, China; wujxcnu@163.com (J.W.); 2140602054@cnu.edu.cn (P.W.); wangfhcnu@126.com (F.W.)

**Keywords:** graphene quantum dots, surface-enhanced Raman spectroscopy, photoluminescence, laser ablation, passivation

## Abstract

Photoluminescence (PL) is the most significant feature of graphene quantum dots (GQDs). However, the PL mechanism in GQDs has been debated due to the fact that the microstructures, such as edge and in-plane defects that are critical for PL emission, have not been convincingly identified due to the lack of effective detection methods. Conventional measures such as high-resolution transmission electron microscopy and infrared spectroscopy only show some localized lattice fringes of GQDs and the structures of some substituents, which have little significance in terms of thoroughly understanding the PL effect. Here, surface-enhanced Raman spectroscopy (SERS) was introduced as a highly sensitive surface technique to study the microstructures of GQDs. Pure GQDs were prepared by laser ablating and cutting highly oriented pyrolytic graphite (HOPG) parallel to the graphite layers. Consequently, abundant SERS signals of the GQDs were obtained on an Ag electrode in an electrochemical environment for the first time. The results convincingly and experimentally characterized the typical and detailed features of GQDs, such as the crystallinity of sp^2^ hexagons, the quantum confinement effect, various defects on the edges, sp^3^-like defects and disorders on the basal planes, and passivated structures on the periphery and surface of the GQDs. This work demonstrates that SERS is thus by far the most effective technique for probing the microstructures of GQDs.

## 1. Introduction

Graphene quantum dots (GQDs), as a new type of zero-dimensional quantum dot, have drawn intense attention due to their unique properties, such as chemical stability, low toxicity, dispersibility in water, controllable size, and wavelength tenability [1,2,3,4,5]. GQDs have great potential for applications in white light-emitting devices [6,7], fluorescent probes [8,9], anticancer therapy [10], biosensors and photocatalysis [11,12,13]. Nano-sized GQDs are composed of sp^2^ hexagonal domains and functional groups around the periphery and surface of the GQD domains. The GQD domains have a band gap that can be tuned by changing their size and shape [14,15,16]. The periphery and surface of the GQD domains can be chemically passivated by electron-donating substituents. Consequently, GQDs exhibit photoluminescence (PL) due to the quantum confinement effect and surface passivation effect [17,18,19,20,21,22].

Thus far, the PL mechanism in GQDs has been debated and therefore requires further clarification. Photoluminescence excitation (PLE) spectra of the GQDs generally show two PL peaks, indicating the two transitions from the triplet energy states of GQDs [17,19,23]. Based on the triplet ground-state carbene model, these two transitions could be considered to be from σ and π to π* due to the electronic conjugate structures, free zigzag sites, and in-plane defects of the GQD domains [19,23]. Another model suggests that the surface state energy levels could be created between π and π* states due to the functional groups on the periphery and surface of the GQDs, resulting in two transitions from π and surface states to the π* state, respectively [17]. Therefore, the PL peaks should be determined by the size, structure and defects of the GQD domains, such as sp^2^ hexagons, edge and in-plane defects, and the chemically passivated structures on the periphery and surface of GQDs. The passivated structures of electron-donating substituents seem to have been partly characterized by Fourier transform infrared (FTIR) spectroscopy, X-ray photoelectron spectroscopy (XPS), etc. [17]. However, the distinct structures of GQD domains, especially the structures of edge and in-plane defects that are always formed and incorporated in sp^2^ hexagons in the synthesis process, have not been reported due to the lack of an effective structure detection method. Only some localized lattice fringes and obscure edge structures of GQD domains, rather than the detailed structures of the defects, were observed by transmission electron microscopy (TEM) and high-resolution transmission electron microscopy (HRTEM) [24,25]. Although the structures of edge and in-plane defects in GQDs have been theoretically presumed to significantly affect the photoluminescence of GQDs [26], little experimental evidence related to the fine structure of defects has been presented, leading to ambiguity.

GQDs have usually been fabricated via top-down cutting routes and bottom-up synthesis methods [27], such as hydrothermal methods [5,28,29,30], enhanced hydrothermal methods [31,32], carbonization [23], acidic exfoliation routes [33], and electrochemical strategies [34,35]. These methods suffer from complex and severe formation processes, including high temperature and concentrated acid environments [33,36,37], inevitably leading to the existence of chemical additives, residues and stabilizers. These impurities in the GQD samples not only complicate the understanding and theoretical simulations of the GQD PL mechanism via the GQD microstructures, but also affect the Raman and surface-enhanced Raman spectroscopy (SERS) measurements of the GQD fine structures, in which pure GQD samples are needed.

Raman spectroscopy is a powerful tool in the structural characterization of graphitic materials [38,39]. However, the Raman spectra of GQDs usually only show a D band at approximately 1350 cm^−1^ and a G band at approximately 1588 cm^−1^ [19,40,41,42,43]. There is a lack of more Raman bands associated with the structure of GQDs. Surface-enhanced Raman spectroscopy (SERS), as a highly sensitive surface technique, can greatly enhance the Raman signals of the molecules adsorbed on noble metal nanoparticles [44,45,46], offering the chance to detect more Raman bands corresponding to the detailed structure of the GQDs.

In this paper, pure GQDs without chemical additives, residues and stabilizers were fabricated for the SERS study by ablating highly-oriented pyrolytic graphite (HOPG) with a pulsed laser beam along the orientation parallel to the graphite layers in deionized water. The SERS spectra of GQDs with abundant peaks were obtained by the chemisorption of GQDs on Ag electrodes in an electrochemical environment. In contrast to the case of two-dimensional large graphene sheets, the D band split into two peaks at 1362 cm^−1^ and 1387 cm^−1^ due to the double resonance effect of graphene. The lower frequency peak related to the edge defects was stronger than the higher frequency peak that was derived from disorders such as sp^3^ structures and the fluctuation of the curvature on the basal planes. The D band was further broadened and blue shifted due to the various defects, disorders and passivated functional groups of the GQDs. The G band was also split into two peaks by symmetry breaking. The additional phonon modes demonstrated that the basal planes of the GQD domains retained the sp^2^ hexagonal structure. The abundance of microstructures in the SERS spectra that were not directly observed with other conventional detection and observation methods indicated that the microstructures of the GQDs could be effectively characterized by SERS. The results contributed to the further understanding of the types of defects and their effects on the PL mechanism in GQDs.

## 2. Materials and Methods

Silver nanoparticle suspensions were prepared by ablating a silver plate in deionized water. The average diameter of the silver nanoparticles was approximately 30 nm. The silver plate was then replaced by highly oriented pyrolytic graphite (HOPG, SPI Supplies Co., West Chester, PA, USA) to fabricate the GQD samples via the ablation of the HOPG along the orientation parallel to the graphite layers in deionized water. Ablation was achieved with a Q-switched Nd: YAG laser with a wavelength of 1064 nm, a repetition rate of 10 Hz, and a pulse width of 6 ns. Consequently, GQDs adequately combined with the silver nanoparticles to form the Ag@GQD samples.

The Ag@GQDs sample was coated on a roughened silver electrode (99.9%) that was placed in a typical electrochemical cell containing a 0.1 M Na_2_SO_4_ solution. A platinum wire and an Ag/AgCl electrode were employed as the counter electrode and reference electrode, respectively. The voltage applied to the working electrode was controlled by a CHI 660A electrochemical instrument (CH Instrument, Bee Cave, TX, USA) The SERS spectra of GQDs on the surface of the working electrode were collected with varying potentials from 0.0 V to −1.2 V.

The morphology of the GQDs was observed by TEM and HRTEM (Tecnai G2 F30, FEI Company, Hillsboro, OR, USA) with an acceleration voltage of 300 kV, accompanied by energy disperse spectroscopy (EDS) measurements. The PL emission spectra were measured with a fluorescent spectrophotometer (F-7000, Hitachi, Tokyo, Japan). The Raman and SERS spectra were measured with a confocal micro-Raman spectrophotometer (Renishaw H 13325, Renishaw PLC, London, UK) and excited at the wavelength of 532 nm. The power of the laser used on the sample was 0.41 mW. Infrared (IR) absorption spectra were recorded with an FTIR spectrometer (60 SXB, Nicolet Instrument. Inc., Madison, WI, USA) using a KBr wafer with a resolution of 4 cm^−1^. XPS spectra were collected using an ESCALAB 250 X-ray photoelectron spectrometer (Thermo Fisher Scientific Inc., Waltham, MA, USA) with a spatial resolution less than 3 μm.

## 3. Results and Discussion

**Figure 1:**
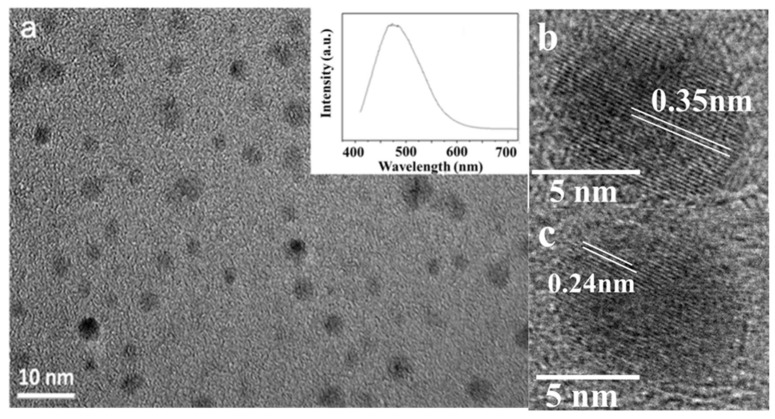
(**a**) TEM images of graphene quantum dots (GQDs) and the photoluminescence (PL) spectrum of GQDs excited at a wavelength of 400 nm (inset); (**b**) and (**c**) High-resolution transmission electron microscopy (HRTEM) images of GQDs.

Figure 1a shows TEM images of GQDs fabricated by ablating HOPG with a pulsed laser beam along the orientation parallel to the graphite layers. GQDs with diameters of 2–6 nm were uniformly and densely dispersed in the products. The HRTEM images show localized lattice fringes of graphene with an in-plane lattice parameter of approximately 0.24 nm (Figure 1b) that can be ascribed to (1120) [23]. The (002) lattice spacing distance was 0.35 nm (Figure 1c), slightly larger than that in bulk graphite (0.336 nm) [19]. The PL emission of GQDs excited at a wavelength of 400 nm showed a peak centered at 473 nm without chemical passivation additives (inset of Figure 1a). Notably, when the HOPG was ablated in the direction perpendicular to the graphite layers, the samples mainly included carbon nanoparticles similar to those that are conventionally achieved when ablating candle soot and graphite powder with a laser [24,47].

**Figure 2:**
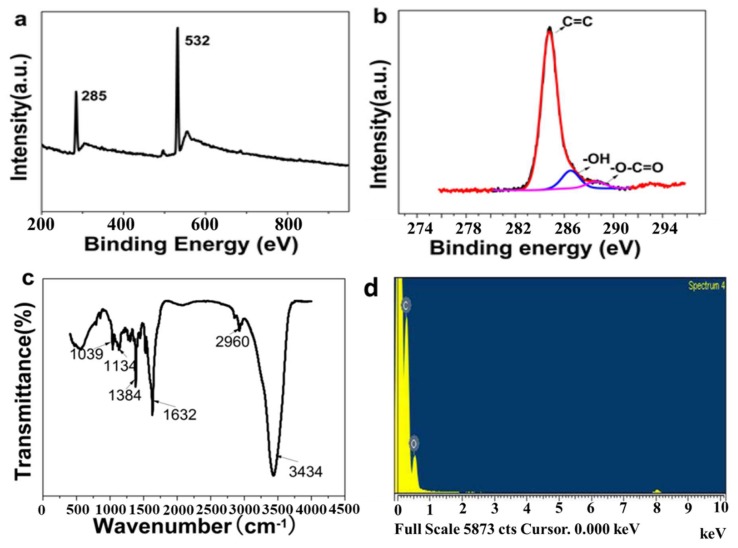
GQDs: (**a**) X-ray photoelectron spectroscopy (XPS) survey spectrum; (**b**) high-resolution XPS C1s spectrum; (**c**) Fourier transform infrared (FTIR) spectrum; (**d**) Energy disperse spectroscopy (EDS) spectrum.

Figure 2 shows the XPS, FTIR and EDS spectra of the GQDs. Overall, these measurements represent an attempt to visualize the structure of the GQDs.

The XPS spectrum shows sharp peaks at binding energies of 285 eV (C1s) and 532 eV (O1s) (Figure 2a). The high-resolution spectrum of the C1s (Figure 2b) shows a wide band consisting of three components at 284.8, 286.5 and 288.7 eV, which are attributed to graphitic, hydroxyl, and carboxylic groups on the periphery and surface of the GQDs, respectively [17,23]. The proportion of the graphitic peak suggests that carbon was the main component of the GQDs.

In the FTIR spectrum (Figure 2c), two typical peaks at 3434 and 1384 cm^−1^ were observed and could be assigned to the stretching vibration and in-plane bending vibration of C–OH, respectively. An intense peak at 1632 cm^−1^ was ascribed to the vibration of C=C. In addition, the absorption of C–O in the carboxylate groups at 1039 and 1134 cm^−1^ and the weak stretching vibration of C–H at 2960 cm^−1^ were observed [17,23]. The IR characterization of GQDs was distinctly different from that of the HOPG [48].

The EDS spectrum shows that the GQDs contain carbon and oxygen (Figure 2d). The atomic ratio of C/O was approximately 9/1, indicating that C was the dominant element in the GQDs.

In summary, the HOPG was ablated with a pulsed laser along the orientation parallel to the graphite layers in order to fabricate single- or few-layer GQDs with high purity and without chemical additives, residues and stabilizers. These impurities existed in the conventionally chemically synthesized GQDs, and their absence was significant for SERS measurements. On the other hand, although some vibrations related to the conjugate structure of C=C and electron-donating substituent groups (C–OH, C=O, C–O–C) were observed, more significant information about the microstructures of the GQD domains, such as in-plane and edge defects, and other defects were not obtained.

**Figure 3:**
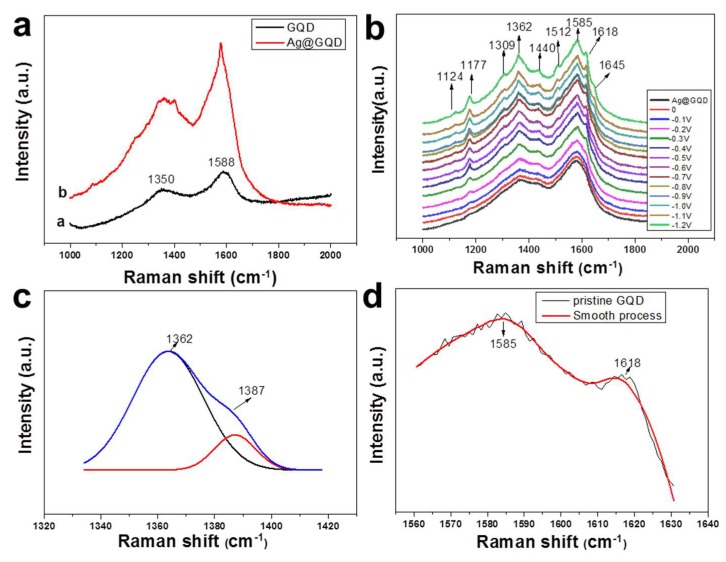
(**a**) Curve a: Raman spectrum of GQDs on silicon, and curve b: Surface-enhanced Raman spectroscopy (SERS) spectrum of GQDs on silver nanoparticles (Ag@GQDs); (**b**) SERS spectra of Ag@GQDs coated on a Ag electrode with changing potentials from 0.0 V to −1.2 V; (**c**) The D band in SERS at −1.2 V (blue) and two fittings (black and red); and (**d**) the G band in SERS at −1.2 V. The excitation wavelength was 532 nm.

In the normal Raman spectrum of the GQDs (curve a of Figure 3a), the G band at 1588 cm^−1^ and the D band at 1350 cm^−1^ were observed [26]. More Raman peaks corresponding to the detailed structures of the GQDs that might be significant for their photoluminescence were not obtained. Curve b of Figure 3a shows the SERS spectrum of GQDs on silver nanoparticles (Ag@GQDs). However, no extra peaks appeared, although the intensities of the D and G bands were enhanced. To further enhance the Raman signals of the GQDs, an electrochemical environment was introduced to tightly combine the Ag@GQD sample and silver electrode. Figure 3b shows the SERS spectra of GQDs on a silver electrode in an aqueous electrochemical environment with varying potentials from 0 V to −1.2 V. Many new peaks appeared and gradually increased in intensity with potential, indicating that nanosized GQDs with enormous edge defects were adsorbed on the Ag electrode, enhancing the SERS signals of the GQDs.

The normal Raman intensity ratio of the D band to the G band was 0.77 (Figure 3a), which was similar to that of other high-quality GQDs [19]. This suggests that during the laser ablation process, more defects were formed on the GQDs. The SERS intensity ratio of the D band to the G band increased with decreasing potential and reached 0.96 at −1.2 V (Figure 3b). Nanosized GQDs had enough edge defects that the numerical ratio of edge defects to in-plane regular hexagons was much higher than that for large graphene sheets, leading to an increase in the relative intensity of the D band. Some defects on the edges of GQD domains can be converted to new defect structures during chemical passivation by electron-donating substituents to form the functional groups, which increase the variety of defects. These functional groups facilitate the chemisorption of GQDs on the Ag electrode and implement charge transfer between GQDs and the Ag surface more readily than the graphitic edge defects of GQD domains. On the other hand, chemical passivation can be strengthened with a decrease in potential, which further increases the charge transfer effect. As a result, the relative intensity of the D band compared to the G band was prominently enhanced. Furthermore, considering that GQDs have various edge defects with complicated structures, the D band in SERS was broadened. Additionally, the calculation also showed that the D band of graphene could be broadened with a decrease in size because of the quantum confinement effect [49,50].

It is noted that with varying potentials from 0.0 V to −1.2 V, the D band was split into a peak and a shoulder (Figure 3b). Lorentzian fitting provides a reasonable approximation of the measured D band, indicating that the D band consisted of two peaks at 1362 and 1387 cm^−1^ (Figure 3c). According to the double resonance theory of graphene, the D band should be composed of two frequencies. The lower frequency is derived from the defects on the edges, and the higher frequency originates from disorders on the basal planes [49,50]. Notably, the intensity of the lower frequency peak of the D band is stronger than that of the higher frequency peak, in contrast to the case of large graphene sheets, in which the lower frequency peak is weaker than the higher frequency peak [51]. GQDs have enormous edge defects. Some of them are chemically passivated by electron-donating substituents, forming functional groups that are more easily chemically adsorbed on Ag electrodes and cause charge transfer between the Ag electrode surface and adsorbed GQDs. This results in the further SERS enhancement of the lower frequency peak. On the other hand, the higher frequency peak, which is related to disorders such as ripple curvature or defects such as carrier doping on the basal planes [39,51], emerges out of the D band as a weak shoulder. Compared to the ripple disorders on the basal planes of large graphene sheets [51], nanosized GQD domains are nearly flat, reducing the fluctuation in curvature. However, the peak at 1309 cm^-1^ that is usually ascribed to sp^3^ hybridization suggests that some graphitic hexagons on the basal planes of GQD domains or armchairs and zigzags on the edges are damaged and converted into defects such as sp^3^ structures during laser ablation and probably induce some degree of curvature disorder [52]. Consequently, the lower frequency peak in the D band is much stronger than the higher frequency peak. This double resonance effect, as a typical feature of graphene, also implies that the basal planes of GQD domains retain the quasi-2D sp^2^ hexagonal structure, although enormous defects exist after laser ablation.

Furthermore, the D band shows a blueshift to 1362 cm^−1^ in SERS compared to its position in normal Raman spectra of graphite, large graphene sheets and GQDs [51,53]. The structures of the edge defects of GQD domains could be redistributed according to the minimum energy principle, leading to a change in the Fermi energy level of the GQDs. The potential applied to the Ag electrode also changed the Fermi energy of the Ag surface. Additionally, the strong quantum confinement effect of GQDs and the charge transfer effect between GQDs and the Ag electrode can tune the lowest unoccupied molecular orbital (LUMO) energy level of GQDs, also leading to a shift in the D band.

For the same reasons the G band also split into two peaks at 1585 and 1618 cm^−1^ (Figure 3d), which can be attributed to the iTO and LO phonon modes (E2g symmetry), respectively, at the Brillouin zone center [54,55]. In a normal Raman spectrum of large graphene sheets, the G band only has a single G Lorentzian peak because of the energy degeneracy of these two optical phonon modes at the Γ point. However, in the SERS of the GQDs, splitting the G band can be induced by the adsorption of GQDs on the Ag electrode, which leads to symmetry breaking.

The mode at 1510 cm^−1^ was ascribed to the iTO phonon near 1/4 ΓK, and the mode at 1650 cm^−1^ was assigned to the LO phonon near ΓK/4, [55,56] which are typical features of graphene, further indicating that the GQD domains not only have disorders and defects such as sp^3^ hybridization on the basal planes, but also basically retain the quasi-2D sp^2^ hexagonal structure. Both new peaks appearing at 1124 and 1177 cm^−1^ were assigned to the vibration of functional groups of C–O, and the peak located at 1440 cm^−1^ was attributed to the C=C vibration, characterizing the passivated functional groups.

In summary, GQDs can be effectively characterized via SERS. The typical features of nanosized GQDs, such as the crystallinity of sp^2^ hexagons, the quantum confinement effect, various edge defects, sp^3^-like defects and disorders on the basal planes of GQDs, and passivated structures on the periphery and surface of GQDs, can be presented by the abundant SERS signals of the GQDs. These results are significant for clearly understanding the PL mechanism in GQDs.

During the synthesis process of GQDs, a number of minute bubbles were produced on the surface of the HOPG as the GQDs were formed by ablating and cutting HOPG with a pulsed laser beam along the orientation parallel to the graphite layers. It is proposed that with laser irradiation, the surface of the HOPG absorbed enough energy over a short time period to increase the temperature in the micro-area. As a result, the graphitic clusters on the laser-ablated micro-area broke out of the HOPG surface. By further overcoming Van der Waals forces, the high-energy graphitic clusters cracked into a single-layer or few-layer nanoscale GQD domains. Simultaneously, water molecules were decomposed to passivate the edges and basal planes of the GQD domains to form the electron-donating substituent groups. GQDs can be stably suspended in water because of the hydrophilicity of the photoinduced functional groups.

## 4. Conclusions

To study the SERS of GQDs, pure GQDs without chemical additives, residues and stabilizers that usually exist in conventionally chemically synthesized GQDs were fabricated by ablating HOPG with a pulsed laser beam along the orientation parallel to the graphite layers in deionized water. To further enhance the SERS signals of GQDs, Ag@GQDs were prepared and adsorbed onto an Ag electrode in an electrochemical environment so that the Ag@GQDs would strongly interact with the Ag electrode at the applied potentials. Consequently, SERS spectra of GQDs with abundant vibrational peaks were obtained. GQDs have various edge defects, including enormous original edge defects and new converted edge defects due to chemical passivation that can be chemisorbed on the Ag electrode to implement charge transfer between the Ag surface and GQDs with potential. This process led to an increase in the intensity of the D band relative to the G band and the broadening and blueshift of the D band. It is noted that D band split into two peaks at 1362 and 1387 cm^−1^, which can be attributed to the double resonance effect of graphene. Due to the enormous edge defects in GQDs, the lower frequency peak related to edge defects was stronger than the higher frequency peak derived from disorders such as sp^3^ structures and the fluctuation of the curvature on the basal planes. This is in contrast to the case of large graphene sheets, in which the lower frequency peak is weaker than the higher frequency peak because the numerical ratio of edge defects to in-plane regular hexagons is much lower than that of GQDs. The splitting of the G band and other phonon modes further demonstrate that GQD domains not only have disorders and defects on the basal planes but also basically retain their quasi-2D sp^2^ hexagonal structure. Some SERS peaks assigned to the vibrations of functional groups were also observed. This work indicates that GQDs can be effectively characterized by SERS. More details of the structures of GQDs that have not been directly observed with other analytical methods are shown in the SERS spectra. The typical features of GQDs—such as the crystallinity of sp^2^ hexagons, the quantum confinement effect, various defects on the edges, sp^3^-like defects and disorders on the basal planes, and passivated structures on the periphery and surface of GQDs—were presented by the abundant SERS signals and were significant for clearly understanding the PL mechanism in GQDs. In the future, these microstructures that are characterized by SERS could be well correlated with the GQD PL effect by theoretical simulation of the GQD PL mechanism via the GQD microstructures.
